# The importance of figures in scientific ‘show and tell’

**DOI:** 10.1242/dmm.050545

**Published:** 2023-11-08

**Authors:** Julija Hmeljak, Kirsty Hooper

**Affiliations:** The Company of Biologists, Bidder Building, Station Road, Histon, Cambridge CB24 9LF, UK

## Abstract

**Summary:** At Disease Models & Mechanisms, we are prioritising and investing in high-quality scientific figures to ensure that the communication of disease biology is accessible and engaging to all.

**Figure:**
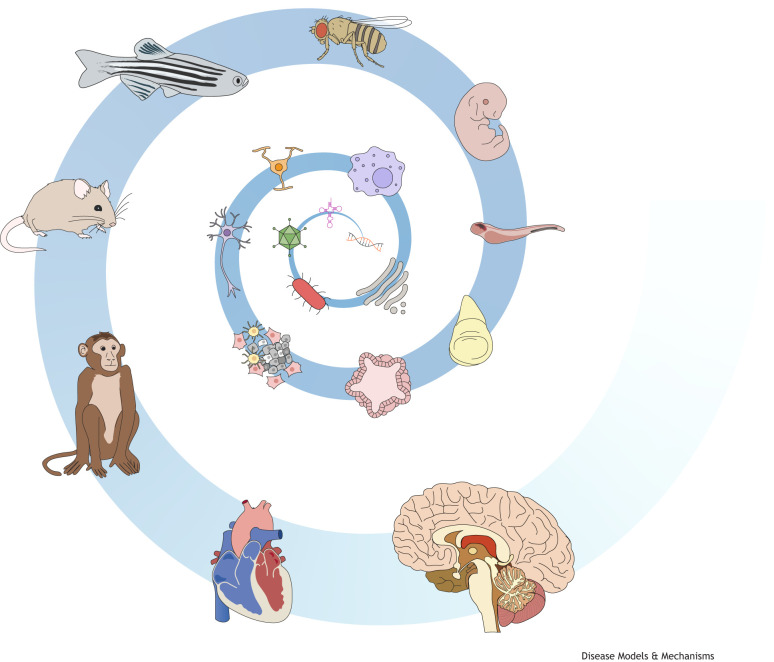


Images are a cornerstone of scientific communication and are crucial in conveying complex biological concepts and functions. The technical art of biological and medical illustration is not a new concept and has been supporting the dissemination of science for centuries, initially through anatomical diagrams; then, as we explored biology at a molecular level, illustrations displayed more granular biological processes. Whilst modern research articles fully rely on figures to communicate results, authors have much more choice when crafting figures for their review-type articles. Whether this freedom represents an exciting or daunting challenge, it is important to remember that, not only does this imagery act as a visual aid for our understanding of biology, but figures are important to engage readers with varying levels of expertise on the topic. Whether perusing a primary research article or a review, it is likely that one of the first things a reader will do is scan through the figures, debating whether it is worth their time to delve deeper into the text. Well-designed figures can capture their attention and deliver complex scientific information with a clarity that plain text alone cannot achieve. If you're already investing your time in writing, it will be worth investing some more in the figures.

In review-type articles, which synthesise vast bodies of knowledge, custom schematics aim to translate complex concepts into accessible at-a-glance visual resources. These figures are an integral part of the article and should be planned early on, such that they seamlessly fit into the article's structure. This will ensure the figures complement, not repeat, concepts already explained in the text. It is important to think about whether you should ‘show’ or ‘tell’ information and what format would be easiest for the reader to digest. At Disease Models & Mechanisms (DMM), we provide guidelines (Box 1) to help invited authors formulate the initial concept and outline of the figures for Reviews, Perspectives, Clinical Puzzles and At a Glance poster articles, which are our most striking example of communicating disease biology through visual means (Martinez Lyons and Boulter, 2021). Combined editorial and peer-review feedback then help authors to optimise their figures before they undergo redrawing by our team of expert illustrators. Their expertise elevates the figures further, creating clear, consistent and polished visual tools. Owing to being published under our CC-BY license, all DMM figures can be shared and reused broadly, for instance, in presentations and teaching.

**Box 1. Tips and tricks for review figure preparation**

1. Structure graphics logically. Each figure tells a story; it might explain a molecular process in a cell, a key disease mechanism or the features of a model system. Keep this story aspect in mind when designing your figure. Use left-to-right/top-to-bottom arrangement so that the information flows logically. The vast majority of readers will look top left first, so this is the recommended starting point of a diagram. Avoid arranging your panels in a circle, unless you are presenting a ‘spider’ diagram with a clear central focus, or you are showing a cycle.

2. Clear hierarchy of the text helps readers perceive the structure of the graphic. Headings and sub-headings should be made clear with larger and/or bold font and this should be consistent throughout the figure. When emphasising any text, bold letters are preferable to underlined, which can be harder to read for people with dyslexia.

3. The format of the font, in general, is very important. It should be large enough to read easily and with good contrast, although black on white can be jarring and difficult to read for people with dyslexia so non-white block backgrounds are preferrable.

4. Avoid overcrowding your image. Remove unnecessary detail to allow the essential elements to stand out. Do not repeat information that you explained in the text.

5. Minimise text – it's a figure for a reason. Instead of including long passages of text, you can move this text to the figure legend or use concise bullet points. Elements should be labelled clearly in the figure but avoid detail overload. You can include a Key if appropriate but keep in mind that this can create extra work for the reader under certain circumstances. If preparing a Key, use concise labels and adequate sizing of the graphic elements, so they are clearly distinguishable and easy to recognise in the figure.

6. Include recognisable ‘landmarks’. For example, if you are showing a signalling pathway, the addition of the cell and nuclear membranes in the background will help orient the readers without overwhelming the imagery.

7. Use colours mindfully. Not every element needs to have its own colour. You can use the same colour to group objects or showcase their relationship, then use different shapes to distinguish between individual elements. Be mindful of colour combinations that may not be accessible to colour-blind readers, such as red-green and blue-yellow.

8. Be consistent within each figure and between figures. Elements that are in aesthetic agreement are easier to be perceived as also being in narrative agreement. This will help readers identify parallels between the figures.

Despite figures being a pillar of scientific communication, they are often an afterthought when preparing manuscripts for publication. This can often be the case for research articles, although most of the principles outlined for review-type articles also apply to figures in research articles. An analysis of 580 articles, published in the top 15 journals across three fields of biology, showed that as low as 2–16% of articles met ‘good practice criteria’ in all of their figures (Jambor et al., 2021). Some of the most prominent misgivings were insufficient labels and annotations, and a lack of accessibility to colour-blind readers. The authors of this analysis offer several examples for how to address these issues, and there is now a multitude of guidance, tools and colour palettes available, to help create clear and accessible figures, some of which are highlighted in Box 1. At DMM, we strongly encourage the use of colour combinations that are suitable for colour-blind readers, and we are reviewing our policies to ensure figures published in our research articles are as accessible as possible.

Our goal is to ensure that the crucial scientific information presented in the figures within research- and review-type articles can be appreciated by the broadest possible readership. As a fully Open Access journal, we strive to communicate high-quality basic and translational research in disease biology to a broad audience of scientists, clinicians and patients (Patton, 2021). The twin pillars of DMM include a strong commitment to excellence and accessibility of all figures that accompany our articles.
